# Disturbed left and right ventricular kinetic energy in patients with repaired tetralogy of Fallot: pathophysiological insights using 4D-flow MRI

**DOI:** 10.1007/s00330-018-5385-3

**Published:** 2018-04-17

**Authors:** Pia Sjöberg, Sebastian Bidhult, Jelena Bock, Einar Heiberg, Håkan Arheden, Ronny Gustafsson, Shahab Nozohoor, Marcus Carlsson

**Affiliations:** 1 Department of Clinical Sciences, Clinical Physiology, Skane University Hospital, Lund University, Lund, Sweden; 2 Department of Biomedical Engineering, Faculty of Engineering, Lund University, Lund, Sweden; 3 Center for Mathematics, Faculty of Engineering, Lund University, Lund, Sweden; 4 Department of Clinical Sciences, Cardiothoracic Surgery, Skane University Hospital, Lund University, Lund, Sweden

**Keywords:** Magnetic resonance imaging, Cine, Tetralogy of Fallot, Pulmonary valve, Heart failure, Heart defects, Congenital

## Abstract

**Objectives:**

Indications for pulmonary valve replacement (PVR) in patients with pulmonary regurgitation (PR) after repaired tetralogy of Fallot (rToF) are debated. We aimed to compare right (RV) and left ventricular (LV) kinetic energy (KE) measured by 4D-flow magnetic resonance imaging (MRI) in patients to controls, to further understand the pathophysiological effects of PR.

**Methods:**

Fifteen patients with rToF with PR > 20% and 14 controls underwent MRI. Ventricular volumes and KE were quantified from cine MRI and 4D-flow, respectively. Lagrangian coherent structures were used to discriminate KE in the PR. Restrictive RV physiology was defined as end-diastolic forward flow.

**Results:**

LV systolic peak KE was lower in rToF, 2.8 ± 1.1 mJ, compared to healthy volunteers, 4.8 ± 1.1 mJ, *p* < 0.0001. RV diastolic peak KE was higher in rToF (7.7 ± 4.3 mJ vs 3.1 ± 1.3 mJ, *p* = 0.0001) and the difference most pronounced in patients with non-restrictive RV physiology. KE was primarily located in the PR volume at the time of diastolic peak KE, 64 ± 17%.

**Conclusion:**

This is the first study showing disturbed KE in patients with rToF and PR, in both the RV and LV. The role of KE as a potential early marker of ventricular dysfunction to guide intervention needs to be addressed in future studies.

**Key Points:**

*• Kinetic energy (KE) reflects ventricular performance*

*• KE is a potential marker of ventricular dysfunction in Fallot patients*

*• KE is disturbed in both ventricles in patients with tetralogy of Fallot*

*• KE contributes to the understanding of the pathophysiology of pulmonary regurgitation*

*• Lagrangian coherent structures enable differentiation of ventricular inflows*

**Electronic supplementary material:**

The online version of this article (10.1007/s00330-018-5385-3) contains supplementary material, which is available to authorized users.

## Introduction

Repair of tetralogy of Fallot (rToF) can result in pulmonary regurgitation (PR) which can cause dilatation of the right ventricle (RV), restrictive RV physiology [1], decreased exercise capacity [2] and predisposes the patient to ventricular arrhythmias and sudden death [3]. The indications and timing of pulmonary valve replacement (PVR) in rToF patients with PR are under debate [4–7]. RV and left ventricular (LV) function are both strongly related to increased mortality, and earlier detection of RV and LV dysfunction may improve selection of patients benefitting from PVR [8].

Four-dimensional (4D)-flow magnetic resonance imaging (MRI) may provide better understanding and early detection of ventricular dysfunction in rToF with PR [9, 10] and the method enables quantification of ventricular kinetic energy (KE) [11]. The KE of blood is defined as the work needed to accelerate a given mass (blood) from rest to its stated velocity. Intracardiac KE has been shown to be disturbed in patients with heart failure, mitral regurgitation and Fontan circulation [12–15]. However, in patients with rToF, there is only one previously published study, which also did not include analysis of diastolic KE [16]. During diastole, PR and tricuspid inflow both contribute to RV KE. To analyse the KE during diastole these flows can be separated using Lagrangian coherent structures (LCS), as previously shown and validated [17]. Turbulent kinetic energy has been used to study the RV of patients with rToF [18]. Turbulent kinetic energy estimates the amount of energy converted into heat because of viscosity and turbulence. Thus, turbulent KE is a different physical entity showing energy loss, compared to KE that reflects the work needed to accelerate intraventricular blood from rest to the velocity at each time point. KE may help explain the pathophysiology of PR and serve as a biomarker in this disease. The aim of this study was to evaluate ventricular kinetic energy during the entire cardiac cycle in patients with rToF with moderate or severe PR compared to controls, in order to describe the pathophysiological effects of PR on the left and the right ventricles.

## Materials and methods

### Study design

Fifteen patients, five female, referred for cardiovascular MRI were prospectively included in the study. Inclusion criteria were rToF, with PR > 20% (diagnosed on previous MRI or echocardiography) and no pulmonary stenosis. Age at inclusion was 29 ± 12 years (mean ± SD). Seven of the patients had a systemic to pulmonary shunt prior to the corrective surgery. Median age at correction was 14 months, range 3–350 months (*n* = 14, age of surgery was uncertain for one patient). Ten patients had a transannular patch inserted. Median time from correction to study inclusion was 18 years (range 9–46 years). One patient had a stenosis of the left pulmonary branch 10 years after corrective surgery, which was treated with a stent 10 years prior to the inclusion. One patient, corrected at age 29, had a reoperation with a conduit 16 years later, 9 years prior to the inclusion. Patients underwent MRI including 4D phase contrast (PC) flow and performed an exercise test with continuous gas analysis to determine peak oxygen uptake (VO_2_ peak) described in detail below [19]. Six of the patients later underwent pulmonary valve replacement (PVR) and had a follow-up MRI 6–12 months after operation with the same protocol as baseline. Indications for PVR were PR fraction ≥ 35%, progressive RV dilatation with RV end-diastolic volume (EDV) ≥ 150 ml/m^2^ and/or symptoms and signs of heart failure. Fourteen healthy volunteers, two female, age 30 ± 7 years, were recruited by advertising at the local institution and underwent the same MRI protocol and were used as controls. Inclusion criteria for controls were normal blood pressure (< 140/90 mmHg) and ECG, no cardiovascular medication and no medical history of cardiovascular or other systemic disease.

The principles of the Declaration of Helsinki were followed and the study was approved by the Regional Ethical Review Board, Lund, Sweden. Written informed consent was obtained from all subjects.

### Cardiac magnetic resonance imaging

Balanced steady-state free-precession (bSSFP) cine images covering the entire heart were acquired using a 1.5-T Achieva MRI (Philips Healthcare,) or a 1.5-T MAGNETOM Aera MRI (Siemens Healthcare). Two-dimensional (2D) PC flow measurements were performed in the ascending aorta and pulmonary artery to measure the effective stroke volume (SV) and PR. Restrictive RV physiology was defined as end-diastolic forward flow in the pulmonary artery [20, 21]. A PC 4D-flow sequence was used to acquire a three-dimensional (3D) volume covering the whole heart. Typical imaging parameters are reported in Table 1. On both scanners prototype 4D-flow sequences for research purpose were used. The 4D sequences have been previously described and validated in vivo and in vitro [22, 23]. The number of time phases acquired was dependent on heart rate and set to the maximum with a preserved segmentation factor/views per segment of 2. Typical acquired matrix size was 80 × 96 × 35 entries. To minimize the potential effect of using two scanners on the study, nine patients and eight controls were examined with the Philips scanner and six patients and six controls with the Siemens scanner. Follow-up studies after PVR were performed on the same scanner as baseline.

**Table 1:** Typical imaging parameters

Sequence parameters	bSSFP CINE	2D Flow	4D Flow
Siemens 1.5 T MAGNETOM Aera MRI			
Flip angle [°]	70	20	8
TE/TR [ms]	1.2/2.7	2.7/4.9	3.5/5.6
Slice thickness [mm]	8	5	Not applicable
Slice gap [mm]	0	Not applicable	Not applicable
Reconstructed spatial resolution [mm^3^]	1.2 × 1.2 × 8	1.6 × 1.6 × 5	3 × 3 × 3
Acquired temporal resolution [ms]	43	10	45
Reconstructed time phases	25	35	40
Gating method	Retrospective ECG	Retrospective ECG	Retrospective ECG
Velocity encoding (VENC) [cm/s]	Not applicable	200	100
Philips 1.5 T Achieva MRI			
Flip angle [°]	60	15	8
TE/TR [ms]	1.4/2.8	3.0/5.2	3.7/6.3
Slice thickness [mm]	8	6	Not applicable
Slice gap [mm]	0	Not applicable	Not applicable
Reconstructed spatial resolution [mm^3^]	1.4 × 1.4 × 8	1.2 × 1.2 × 6	3 × 3 × 3
Acquired temporal resolution [ms]	47	10	50
Reconstructed time phases	30	35	40
Gating method	Retrospective ECG	Retrospective ECG	Retrospective ECG
Velocity encoding (VENC) [cm/s]	Not applicable	200	100

*bSSFP* balanced steady-state free-precession, *2D* 2-dimensional, *4D* 4-dimensional, *TE* echo time, *TR* repetition time

### Image analysis and calculation of kinetic energy

Segment software (http://segment.heiberg.se) with an in-house developed module for 4D-flow analysis of KE was used [24]. First-order phase background correction [25] and phase unwrapping was performed. For each voxel the velocity magnitude of the 3D velocity vector was computed.

Delineations of the endocardium were drawn manually over the cardiac cycle in the cine short-axis stack to calculate EDV, end-systolic volume (ESV) and SV. The delineations were transferred to the 4D-flow data set. KE for each voxel was calculated as $\mathrm{KE}=\frac{\mathrm{m}{\mathrm{v}}^2}{2}$, where *m* is mass of the voxel, calculated as the density of blood, 1.05 g/cm^3^, multiplied by the volume of the voxel, and *v* is the velocity in the voxel. KE over all voxels in the ventricles was calculated for all time phases of the cardiac cycle. Systolic and diastolic peak KE was defined as the highest value during systole and diastole, respectively.

**Fig. 1:**
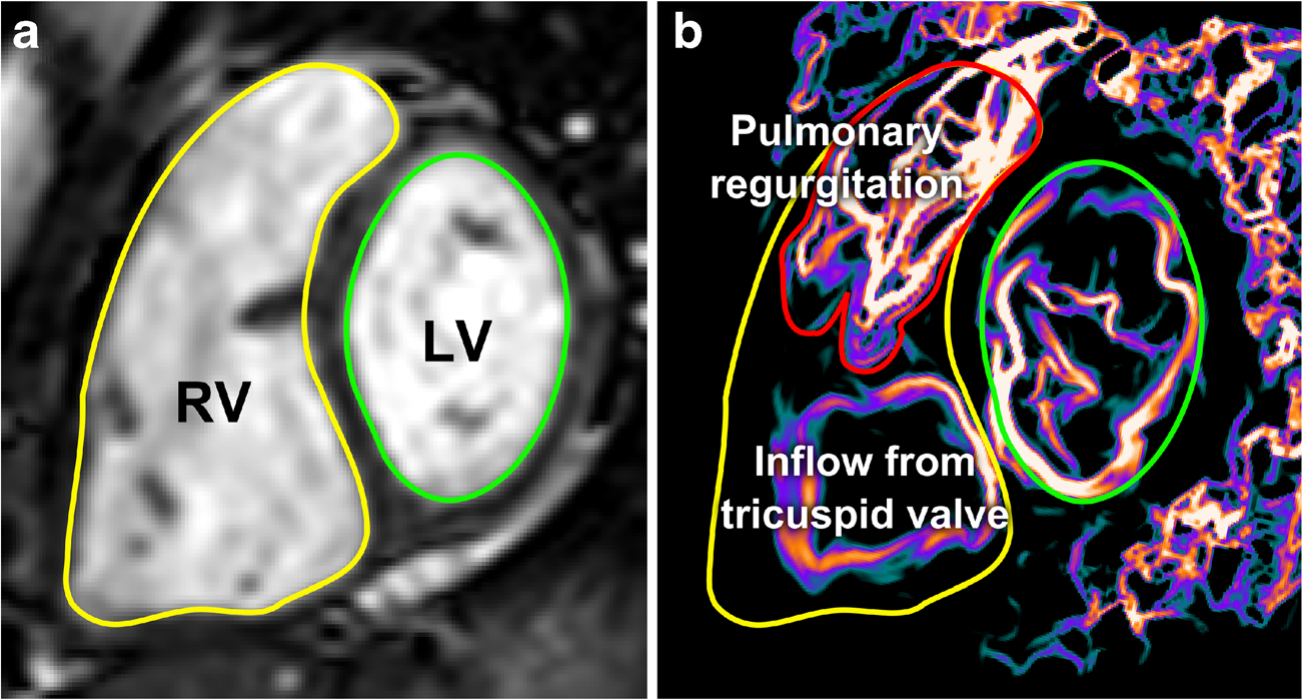
**a** SSFP short-axis view with the right ventricle (RV, outlined in yellow) and the left ventricle (LV) outlined in green. **b** Same view with Lagrangian coherent structures (LCS) at the time of diastolic peak kinetic energy, visualizing the pulmonary regurgitation (outlined in red) and inflow to the right ventricle. LCS define boundaries of flow fields and thereby give a ring shape when a jet is directed towards or from the viewer in this image plane, e.g. the inflow to the RV

LCS were computed in the short-axis orientation at the time of diastolic peak KE in order to find regions of PR and to calculate how much PR contributed to the KE of the RV, Fig. 1 [17].

The ratio between systolic/diastolic peaks was calculated to determine if the pattern in the LV and RV differed between patients and controls and if restrictive physiology had an impact on the KE pattern. KE data were linearly interpolated in time to display the average KE over time in patients and controls independent of different heart rates as shown in Fig. 4 [11].

KE was analyzed as absolute values but also indexed to planimetrically calculated SV (EDV-ESV) to be able to compare patients with different heart sizes or valvular insufficiencies and to eliminate the possibility of a larger SV, caused by the PR in rToF patients, explaining potential differences. There have been different ways of indexing KE in earlier publications and therefore we report the values in this study using five different indexing methods.

### Exercise performance

Patients performed a maximal exercise test with continuous gas analysis (Carefusion, Oxycon Pro, Jaeger) on a cycle ergometer (939 E, Monark). An individualized protocol with a 1-min rest phase, 2-min reference phase on no or low resistance, followed by a ramp with incremental workload until maximal exhaustion was used. Patients were encouraged to continue until respiratory exchange ratio was 1.1 or more, to ensure maximal exertion. Reference values for VO_2_ peak were obtained from the SHIP study [19]. Twelve-lead ECG was recorded continuously and blood pressure was obtained every minute.

### Statistical analysis

Statistical analysis was performed using GraphPad (v6.04, La Jolla). Values are presented as means ± SD. Differences in KE between rToF patients and healthy volunteers were assessed using the Student *t* test and differences before and after PVR using paired *t* test. Cohen’s kappa was analyzed for the relation of systolic/diastolic peak KE and restrictive RV physiology. Correlations were analyzed using Spearman correlation test. Results with a *p* value less than 0.05 were considered statistically significant.

## Results

Results of the volumetric measurements in patients (*n* = 15, five female, age 29 ± 12 years) and controls (*n* = 14, two female, age 30 ± 7 years) are shown in Table 2.

**Table 2:** Characteristics and volumetric measurements in patients and controls

Mean ± SD	Patients with rToF (*n* = 15)	Controls (*n* = 14)	
Age (years)	29 ± 12	30 ± 7	*p* = 0.71
Gender (male/female)	10:5	12:2	
BSA (m^2^)	1.9 ± 0.2	2.0 ± 0.2	*p* = 0.11
LVEDV (ml)	154 ± 25	195 ± 30	*p* < 0.004
LVEDV/BSA (ml/m^2^)	82 ± 11	97 ± 10	*p* = 0.00007
LVESV (ml)	71 ± 16	81 ± 17	*p* = 0.097
LVESV/BSA (ml/m^2^)	38 ± 7	40 ± 7	*p* = 0.29
LVEF (%)	54 ± 6	57 ± 5	*p* = 0.16
RVEDV (ml)	271 ± 73	199 ± 41	*p* < 0.0001
RVEDV/BSA (ml/m^2^)	145 ± 23	99 ± 14	*p* < 0.0001
RVESV (ml)	155 ± 51	88 ± 22	*p* < 0.0001
RVESV/BSA (ml/m^2^)	83 ± 19	55 ± 7	*p* < 0.0001
RVEF (%)	41 ± 5	56 ± 6	*p* < 0.001
PRF (%)	36 ± 13	-	
PR (ml)	51 ± 16	-	
HR (bpm)	71 ± 10	60 ± 9	*p* = 0.006

*rToF* repaired tetralogy of Fallot, *BSA* body surface area, *LVEDV* left ventricular end-diastolic volume, *LVESV* left ventricular end-systolic volume, *LVEF* left ventricular ejection fraction, *RVEDV* right ventricular end-diastolic volume, *RVESV* right ventricular end-systolic volume, *RVEF* right ventricular ejection fraction, *PRF* pulmonary regurgitation fraction, *HR* heart rate

### Absolute and indexed KE values

**Fig. 2:**
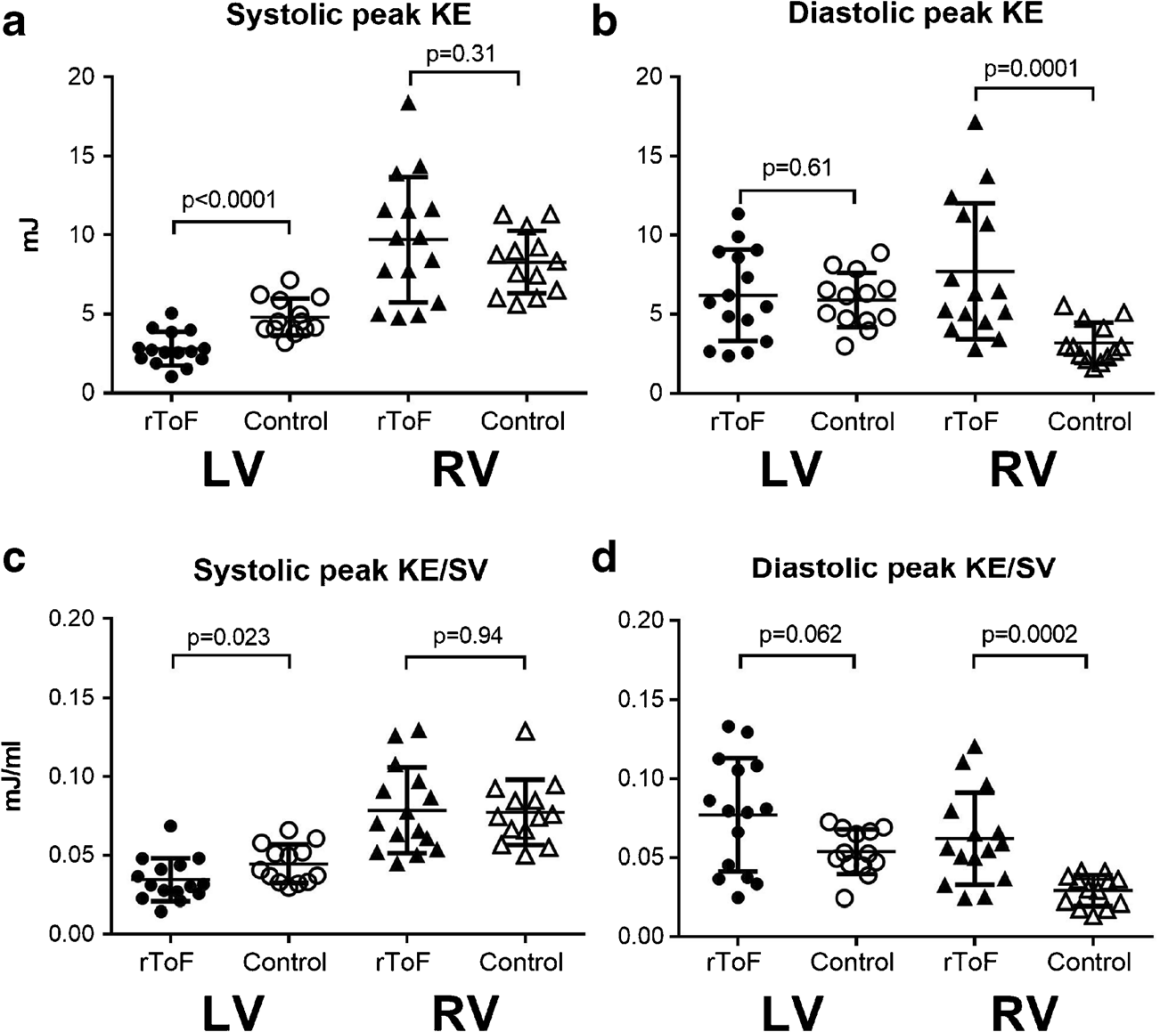
Comparison of peak kinetic energy (KE) (**a**, **b**) and peak KE indexed to stroke volume (SV) (**c**, **d**) between rToF patients and controls for both left ventricle (LV) and right ventricle (RV). **a**, **c** Systolic KE and **b**, **d** diastolic KE. Bar and whiskers show mean ± SD

Systolic peak KE in the LV was lower in rToF compared to controls. Conversely, in the RV diastolic peak KE was higher than in controls. The differences between rToF and controls remained when indexing for SV, Fig. 2. The absolute and indexed KE values are shown in Table 3 together with different indexations used for KE previously.

**Table 3:** Ventricular peak kinetic energy indexed to planimetric stroke volume, cardiac output, body surface area, and inverted to aortic and pulmonary flow for left and right ventricle patients with repaired tetralogy of Fallot and controls

	Mean ± SD rToF, *n* = 15 Controls, *n* = 14	Peak KE (mJ)	Peak KE/SV (mJ/ml)	Peak KE/ systemic CO	Peak KE/BSA (mJ/ml/m^2^)	*Q*/peak KE (ml/cycle/mJ)	*Q*/peak KE/BSA (ml/cycle/mJ/m^2^)
LV	rToF systole	2.8 ± 1.1 ****	0.03 ± 0.01 *	0.59 ± 0.23 *	1.5 ± 0.6 ***	28.8 ± 13.3 NS	15.6 ± 7.6 NS
	Controls systole	4.8 ± 1.1	0.04 ± 0.01	0.75 ± 0.17	2.4 ± 0.5	24.8 ± 6.3	12.5 ± 3.6
	rToF diastole	6.2 ± 2.9 NS	0.08 ± 0.04 NS	1.18 ± 0.56 NS	3.4 ± 1.7 NS	14.5 ± 7.6 *	7.7 ± 4.1 *
	Controls diastole	5.6 ± 1.9	0.05 ± 0.02	0.88 ± 0.33	2.8 ± 1.0	23.3 ± 11.5	11.6 ± 5.5
RV	rToF systole	9.7 ± 4.0 NS	0.08 ± 0.03 NS	1.98 ± 0.74 *	5.2 ± 1.8 NS	9.0 ± 4.4 **	4.9 ± 2.5 *
	Controls systole	8.2 ± 1.9	0.08 ± 0.02	1.30 ± 0.38	4.1 ± 0.9	13.9 ± 3.3	7.0 ± 1.9
	rToF diastole	7.7 ± 4.3 ***	0.06 ± 0.03 ***	1.49 ± 0.79 ****	4.0 ± 2.0 ****	7.9 ± 4.3 ****	4.3 ± 2.2 ****
	Controls diastole	3.1 ± 1.3	0.03 ± 0.01	0.49 ± 0.24	1.6 ± 0.7	41.1 ± 17.3	20.4 ± 8.1

*KE* kinetic energy, *SV* planimetric stroke volume, *CO* cardiac output, *BSA* body surface area, *Q* flow, *rToF* repaired tetralogy of Fallot, *LV* left ventricle, *RV* right ventricle

*p < 0.05, **p < 0.01, ***p < 0.001, ****p < 0.0001 when comparing rToF to controls

**Fig. 3:**
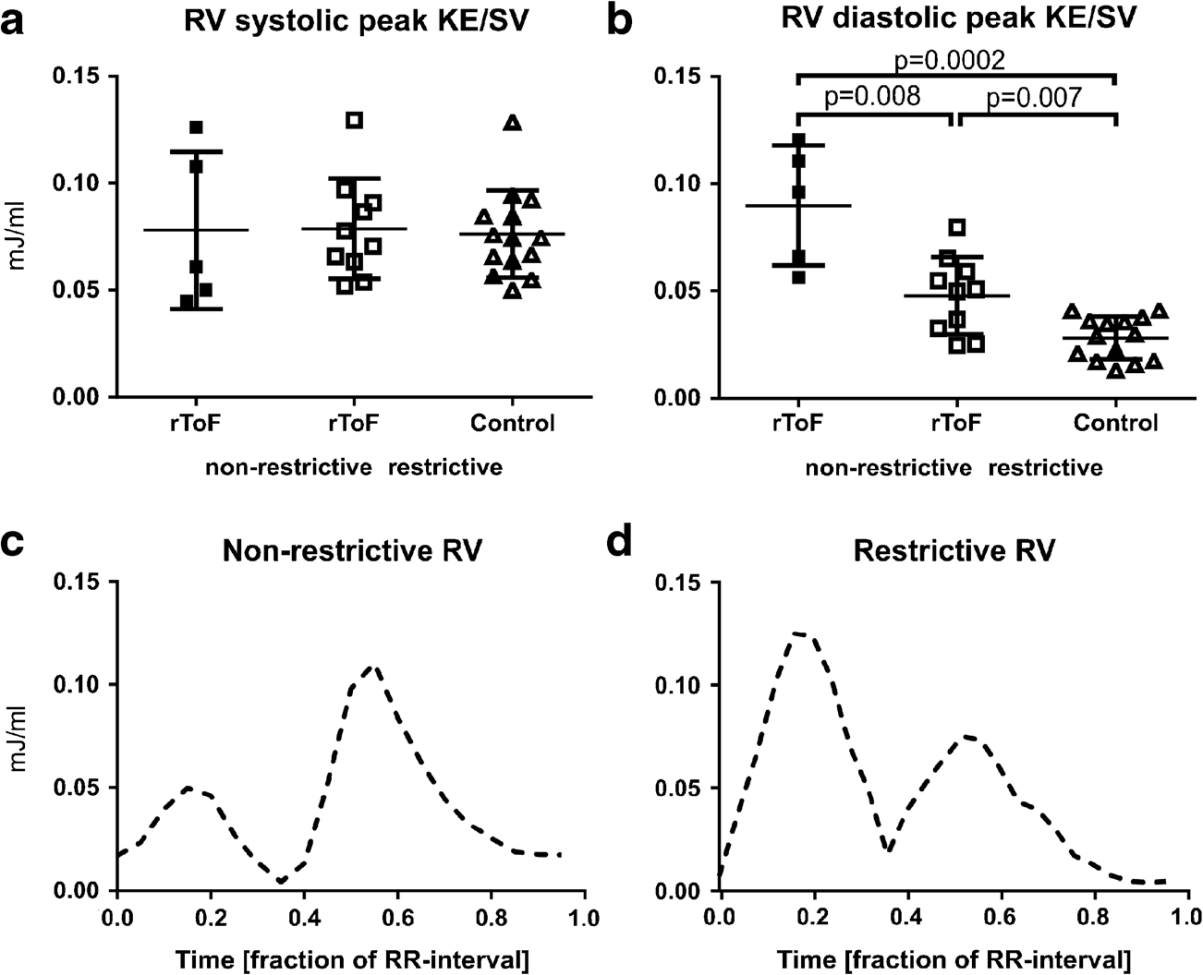
Comparison of systolic and diastolic peak kinetic energy (KE) indexed to stroke volume (SV) in the right ventricle (RV) of patients with repaired tetralogy of Fallot (rToF) with non-restrictive right ventricular physiology, with restrictive right ventricular physiology and controls. Bar and whiskers show mean ± SD. **a** Results for systolic peak KE/SV with no difference between the groups. **b** In diastole, rToF has higher KE/SV than controls, but the difference is more pronounced in rToF with non-restrictive right ventricular physiology than with restrictive right ventricular physiology. The bottom panels show two patient examples of kinetic energy (KE) indexed to stroke volume (SV) throughout the cardiac cycle. **c** Patient with non-restrictive right ventricular (RV) physiology and **d** patient with restrictive RV physiology. Patients with non-restrictive RV physiology had lower systolic peak KE compared to diastolic peak KE resulting in a ratio of peak systolic to peak diastolic KE of ≤ 1 whereas patients with a restrictive RV physiology had a ratio > 1

The increase in diastolic KE was most pronounced in patients with non-restrictive RV physiology, Fig. 3a, b. This difference was seen even though the degree of PR in patients with restrictive and non-restrictive RV physiology was similar (39 ± 11% vs 39 ± 6%, *p* = 0.9).

There was a moderate positive correlation between systolic peak KE and RVEDV in patients with rToF (*R* = 0.54, *p* = 0.04) and a strong positive correlation between diastolic peak KE and RVESV (*R* = 0.74, *p* = 0.002). KE was primarily located in the PR volume at the time of diastolic peak KE, 64 ± 17 %, *n* = 14, and there was a moderate positive correlation between the KE located in the PR fraction and the PR volume, *R* = 0.56, *p* = 0.04. Diastolic peak KE in the entire RV, on the other hand, did not correlate with PR volume (*p* = 0.12).

The differentiation of tricuspid inflow and PR flow is shown in Supplemental Movie 1.

There was no difference (*p* = 0.20) in the diastolic peak KE in the RV outside the PR volume in rToF (2.5 ± 1.1 mJ, indexed to net SV 0.032 ± 0.014 mJ/ml) and the diastolic peak KE in controls (3.1 ± 1.3 mJ , indexed to SV 0.028 ± 0.01 mJ/ml).

### Systolic/diastolic KE ratio

**Fig. 4:**
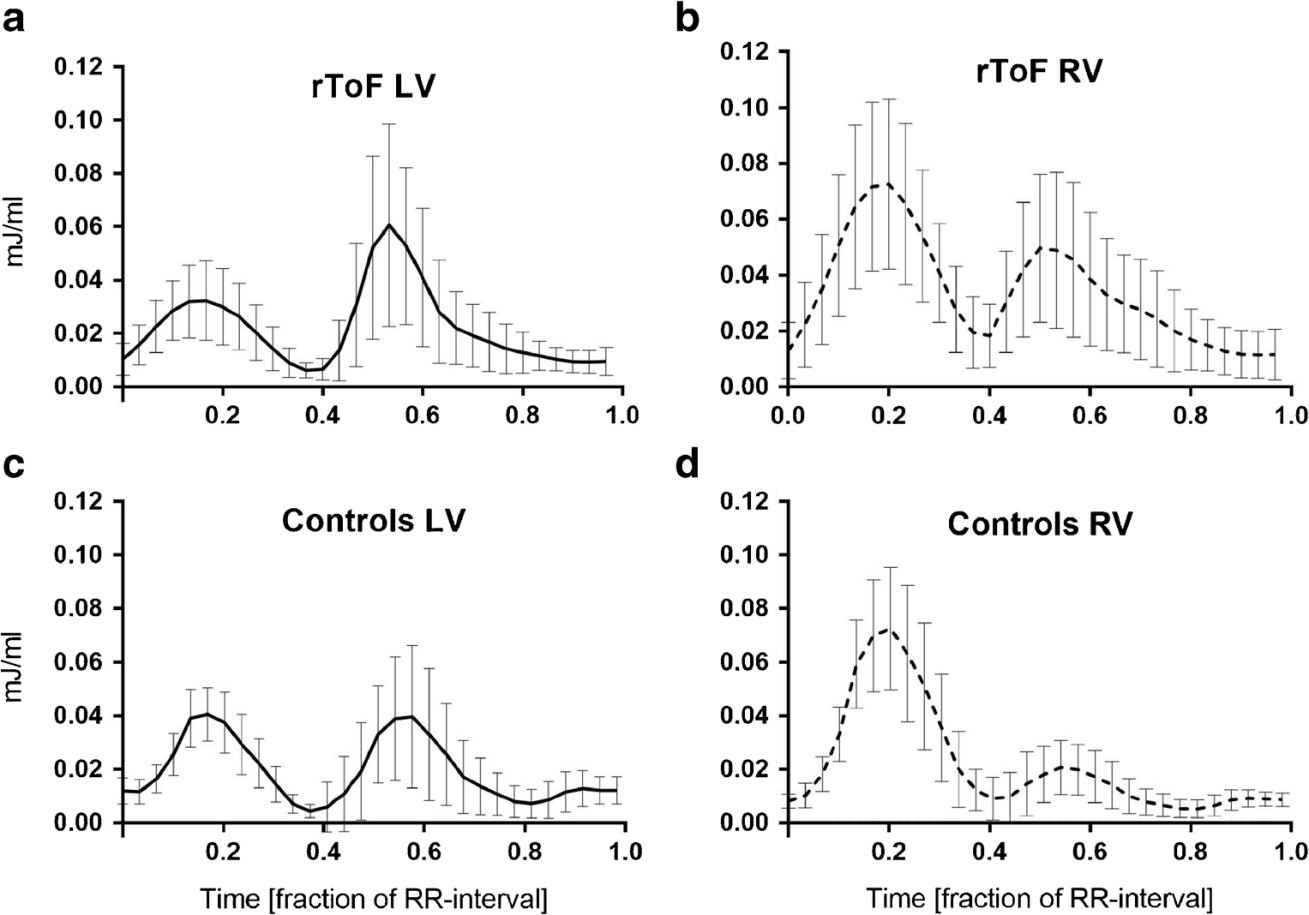
Mean kinetic energy (KE) throughout the cardiac cycle, indexed to stroke volume (SV) with error bars showing the standard deviation. **a**, **b** Results from the left (LV) and right (RV) ventricles of patients with repaired tetralogy of Fallot (rToF). **c**, **d** Results from the LV and RV of controls

The KE/SV throughout the cardiac cycle in the LV and RV of rToF and controls is shown in Fig. 4. The ratio of systolic/diastolic peak KE was lower in rToF for both the LV (0.5 ± 0.3) and the RV (1.5 ± 0.9), compared to controls (1.0 ± 0.4, *p* = 0.0031 for LV and 3.0 ± 1.1, *p* = 0.0006 for RV). Four of five patients with non-restrictive RV physiology had RV systolic/diastolic KE ratio ≤ 1 and the remaining patient had an RV KE ratio of 1.1, whereas a systolic/diastolic KE ratio > 1 was seen in all 10 patients with restrictive RV (Cohen’s kappa 0.84), Fig. 3c, d.

### Exercise performance

Mean peak VO_2_ was 30 ± 10 ml/min/kg (*n* = 13), equivalent to 86 ± 19 % of the predicted value. There was no correlation between peak VO_2_ and PR volume, (*R* = − 0.18, *p* = 0.56), nor were there any correlations between peak VO_2_ and systolic or diastolic peak KE/SV in either RV (*R* = − 0.12, *p* = 0.70; *R* = − 0.03, *p* = 0.92) or LV (*R* = − 0.19, *p* = 0.54; *R* = 0.24, *p* = 0.44). Furthermore, no correlation was found between peak VO_2_ and EDV, ESV, SV or EF in either RV (*R* = − 0.39, *p* = 0.19; *R* = − 0.41, *p* = 0.16; *R* = − 0.32, *p* = 0.28; *R* = 0.16, *p* = 0.61) or LV (*R* = − 0.044, *p* = 0.89; *R* = − 0.10, *p* = 0.73; *R* = 0.047, *p* = 0.88; *R* = 0.17, *p* = 0.59).

### Follow-up after pulmonary valve replacement

 RVEDV decreased from 316 ± 48 ml to 230 ± 43 ml at follow-up after surgery, RVEDV/BSA from 164 ± 18 ml/m^2^ to 116 ± 18 ml/m^2^, RVESV from 188 ± 36 ml to 144 ± 35 ml and RVESV/BSA from 97 ± 3 ml/m^2^ to 73 ± 15 ml/m^2^ (*n* = 6).

Patients after PVR had lower systolic peak KE in both LV and RV, but no difference during diastole, compared to controls, Table 4,

**Table 4:** Peak kinetic energy in patients with repaired tetralogy of Fallot before and after pulmonary valve replacement compared to controls

	Mean ± SD	rToF before PVR *n* = 6	rToF after PVR *n* = 6	Controls *n* = 14
LV	Systolic KE (mJ)	2.7 ± 1.3	3.1 ± 0.8	4.8 ± 1.1^††^
	Systolic KE/SV (mJ/ml)	0.04 ± 0.02	0.03 ± 0.01	0.04 ± 0.01
	Diastolic KE (mJ)	4.4 ± 2.4	4.7 ± 2.1	5.6 ± 1.9
	Diastolic KE/SV (mJ/ml)	0.06 ± 0.04	0.06 ± 0.03	0.05 ± 0.02
RV	Systolic KE (mJ)	8.8 ± 3.7	4.5 ± 1.8	8.2 ± 1.9^†††^
	Systolic KE/SV (mJ/ml)	0.07 ± 0.03	0.06 ± 0.03	0.08 ± 0.02^†^
	Diastolic KE (mJ)	8.5 ± 5.3	2.8 ± 1.1*	3.1 ± 1.3
	Diastolic KE/SV (mJ/ml)	0.07 ± 0.03	0.04 ± 0.02	0.03 ± 0.01

*rToF* repaired tetralogy of Fallot, *PVR* pulmonary valve replacement, *LV* left ventricle, *RV* right ventricle, *KE* kinetic energy

*p < 0.05 rToF before vs after PVR

^†^*p* < 0.05, ^††^*p* < 0.01, ^†††^*p* < 0.001 rToF after PVR vs controls

**Fig. 5:**
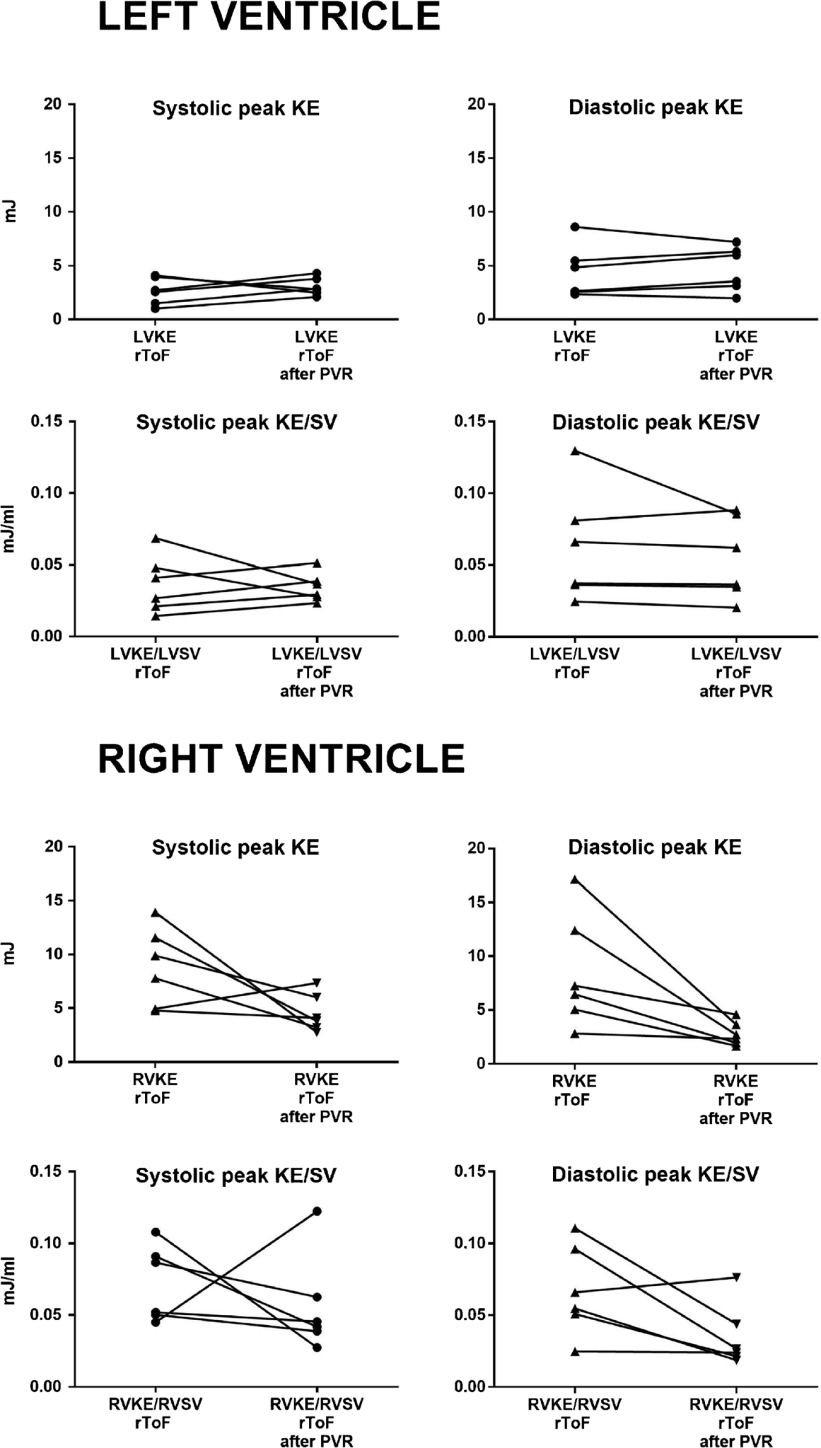
Comparison on individual basis of kinetic energy (KE) and KE indexed to stroke volume (SV) between rToF patients before and after pulmonary valve replacement (PVR). The upper panels show the left ventricle (LV) and the lower panels show the right ventricle (RV). The systolic and the diastolic values are shown in the left and the right column, respectively. All patients but one had a decrease of both systolic and diastolic KE the RV. The rise in KE in one patient is likely explained by the surgical removal of a widening patch in the RV outflow tract at the time of PVR

**Fig. 6:**
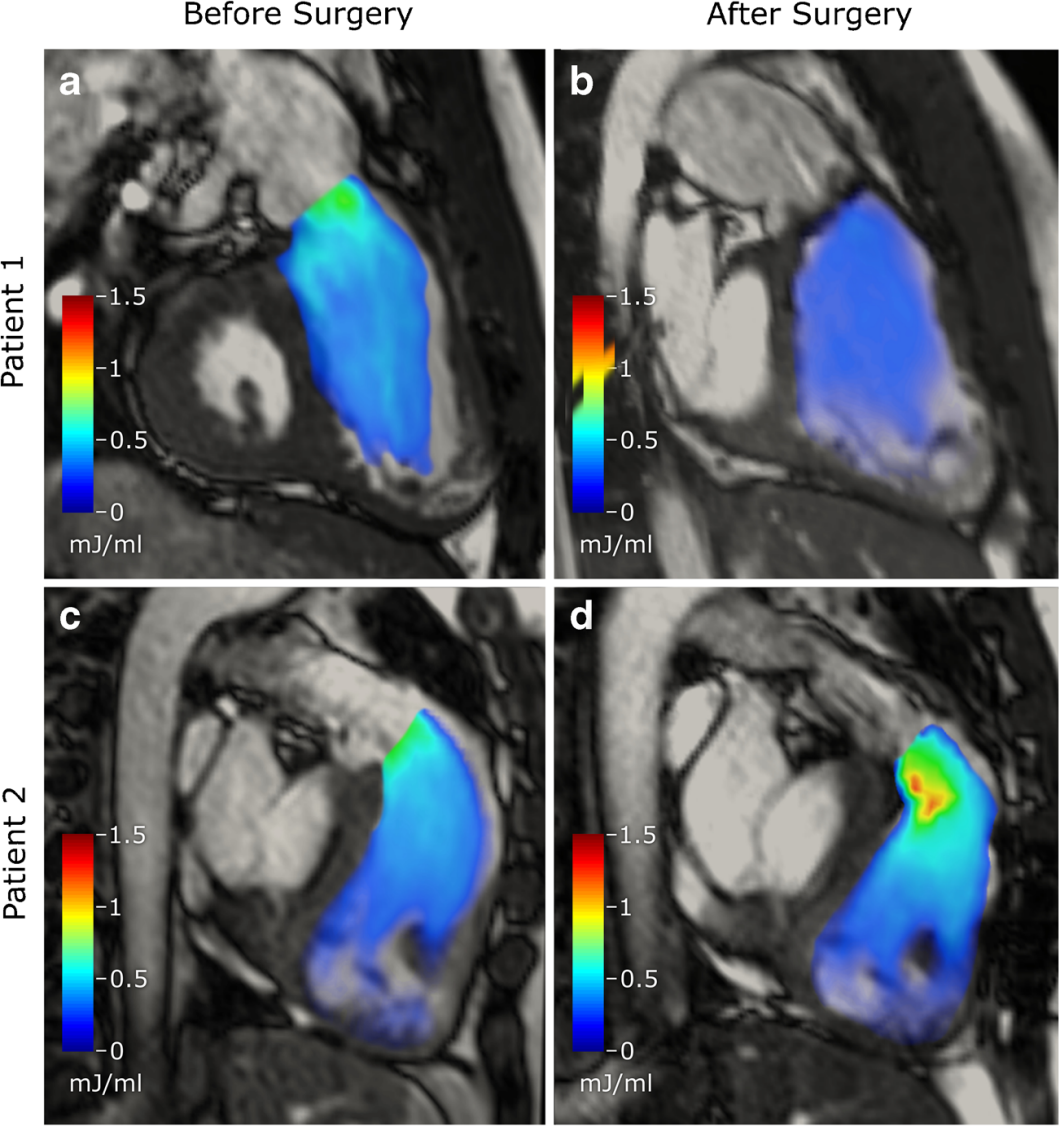
Visualization of kinetic energy during systole in two patients with tetralogy of Fallot before (left column) and after (right column) pulmonary valve replacement (PVR). Patient 1 shows a decrease in systolic KE in the right ventricle (RV) after operation (**b**). **c**, **d** Patient who had a widening patch in the RVOT removed at the time of PVR, which led to higher systolic KE in the RV after surgery than before

All but one patient had decreased peak KE in the RV after PVR, Fig. 5 and Table 4. Visualizations of KE during systole in the RV before and after PVR in two patients are shown in Fig. 6.

## Discussion

This is, to our knowledge, the first study that quantifies KE during the entire cardiac cycle in patients with rToF and PR. Our main findings are (1) Patients with rToF and PR with preserved LV global function have decreased LV systolic peak KE compared to controls. (2) RV diastolic peak KE is increased in patients compared to controls as a result of the KE in the PR volume, but RV systolic KE is not different from controls even though flow volumes are much increased. (3) Patients with restrictive RV physiology have less increased KE compared to patients with non-restrictive RV physiology. (4) After surgical PVR systolic KE was more similar to controls in both ventricles. (5) 4D-flow and LCS enable differentiation of flows in patients with valvular regurgitation.

The decreased systolic peak KE in the LV in patients is interesting as LV strain has been found to be decreased and regional myocardial velocities altered in rToF patients with preserved LVEF [26, 27] and LV function is a strong predictor of outcome in these patients [28]. The difference in KE cannot be attributed to a decreased SV as the difference remains when indexing for SV. The reason for lower systolic peak KE is speculative, but might partly be caused by decreased preload, i.e. an under-filling of the LV due to the RV impairment. This hypothesis is supported by an experimental study that showed that PR leads to decreased longitudinal contribution to RVSV and that the degree of decreased longitudinal contribution to RVSV had a very high correlation with decrease in pulmonary capillary wedge pressure [29]. Septal dyssynchrony with septal movement towards the RV in systole, as described by Stephensen et al. [1] in patients with rToF and also found in the animal model of PR [29], is another factor that may influence the KE in the LV.

The lack of difference in absolute RV systolic peak KE between rToF and controls may seem surprising as the systolic RVSV is higher compared to controls as a result of the PR. KE is determined by mass (volume of blood) and velocity squared. A larger volume passing a fixed RV outflow tract (RVOT) raises KE which explains the functional pressure gradient between the RV and the pulmonary artery seen during increased flows [30]. A possible explanation for our result is that since the RVOT in our group of rToF is wider than in controls a larger volume can pass without increased velocity. Our findings support those of Jeong et al. who found that there was no significant difference between systolic RV KE in rToF compared to healthy volunteers [16]. After PVR there was a non-significant trend toward decreased systolic peak KE in the RV due to the lower volume passing the RVOT. The explanation why the systolic peak KE was even lower compared to the control group postoperatively is presumably the wider RVOT in patients than controls. In one patient systolic peak KE increased after PVR likely because a widening patch in the RVOT was removed at the time of PVR, Fig. 6. Thus, even if the SV postoperatively was lower, the outflow tract in this patient narrowed with increase in velocities causing higher KE.

Diastolic peak KE in the RV was higher in patients than in controls, independent of indexation to SV. Pulmonary regurgitation caused this increase, since the major part of the KE was within the PR volume and the diastolic KE also decreased after PVR.

We showed a positive correlation between RV volumes and KE and this is expected as KE is a product of mass, in this case the RV volume, and the squared velocity of the blood. However, KE and volumes are not simply exchangeable as shown by the lack of correlation between diastolic KE and PR volume. The correlation between PR volume and KE was only demonstrated when separating the KE in the inflowing PR volume.

In the present study, the degree of PR was similar in patients with restrictive and non-restrictive RV physiology, but patients with restrictive RV physiology had lower diastolic KE and different ratio between systolic/diastolic KE compared to non-restrictive patients. The pathophysiological explanation may be that a restrictive RV with less compliance decreases the velocities of the flow through the tricuspid valve and in the PR and thus decreases the KE even if the PR volume is similar. The reasons for systolic to diastolic KE ratio < 1 in a non-restrictive RV and a healthy LV are different. The non-restrictive compliant RV allows high velocities in the PR volume, leading to high diastolic KE, whereas in the healthy LV the high diastolic KE is caused by ventricular suction of blood from the left atrium.

A previous study found that the degree of restrictive physiology had the strongest correlation with predicted VO_2_ and that RV volumes did not correlate with predicted VO_2_ [21]. The difference in KE between restrictive and non-restrictive RV physiology is a potential explanation for the differences in exercise capacity. We found a decreased predicted peak VO_2_ with a large variation, as expected in this patient group [2]. However, no correlation was seen between peak VO_2_ and KE in either LV or RV, but this may be due to the low number of patients. Larger patient cohorts with 4D-flow examination and measurement of peak VO_2_ are needed to further investigate this possible relationship.

In this study we adopted a novel approach of using LCS calculated from 4D-flow to differentiate inflow to the RV from the tricuspid valve and the PR. This method can be used in future studies describing the pathophysiology of other valvular regurgitation using KE.

All subjects in this study were investigated at rest. Studies have shown that KE accounts for little of the external work in healthy controls at rest, but the proportion increase to 3% in the LV and 24% in the RV during exercise [31, 32]. What happens to the KE in patients with rToF and PR is not known but the PR has been shown to decrease during exercise [33].

### Limitations

The patient population size was rather small, in particular when studying the effect of PVR. Nonetheless, differences between patients and controls were found but future prospective outcome studies are needed to show the clinical impact of these findings.

## Conclusion

4D-flow MRI show disturbed kinetic energy in patients with rToF and PR not only in the directly affected RV but also in the LV where the global function is seemingly preserved. 4D-flow KE may be key to understanding why LV function is affected in rToF patients even when global function is normal and explain the strong prognostic impact of LV function in these patients. The clinical importance of adding 4D-flow data to an echocardiographic examination and in particular if the information from 4D-flow alters clinical decision-making and outcome will be of interest in future studies.

## Electronic supplementary material

ESM 1 (MP4 550 kb)

## Abbreviations

3D: Three dimensional
4D: Four dimensional
CO: Cardiac output
EDV: End-diastolic volume
ESV: End-systolic volume
KE: Kinetic energy
LCS: Lagrangian coherent structures
LV: Left ventricle
*m*: Mass
MRI: Magnetic resonance imaging
PC: Phase contrast
PR: Pulmonary regurgitation
PVR: Pulmonary valve replacement
rToF: Repair of tetralogy of Fallot
RV: Right ventricle
RVOT: Right ventricular outflow tract
SV: Stroke volume
*v*: Velocity
VO_2_ peak: Peak oxygen uptake
